# Myrcene: A Natural Compound Showing Anticancer Activity in HeLa Cells

**DOI:** 10.3390/molecules28186728

**Published:** 2023-09-21

**Authors:** Luca Pincigher, Francesca Valenti, Christian Bergamini, Cecilia Prata, Romana Fato, Riccardo Amorati, Zongxin Jin, Giovanna Farruggia, Diana Fiorentini, Natalia Calonghi, Chiara Zalambani

**Affiliations:** 1 Department of Pharmacy and Biotechnology (FaBiT), University of Bologna, Via Irnerio 48, 40126 Bologna, Italy; pincigherluca@gmail.com (L.P.); francesca.valenti8@unibo.it (F.V.); christian.bergamini2@unibo.it (C.B.); cecilia.prata@unibo.it (C.P.); romana.fato@unibo.it (R.F.); giovanna.farruggia@unibo.it (G.F.); chiara.zalambani2@unibo.it (C.Z.); 2 Department of Chemistry “G. Ciamician”, University of Bologna, Via Gobetti 83, 40129 Bologna, Italy; riccardo.amorati@unibo.it (R.A.); zongxin.jin@studio.unibo.it (Z.J.); 3 National Institute of Biostructures and Biosystems, Via delle Medaglie d’Oro 305, 00136 Rome, Italy

**Keywords:** monoterpenes, myrcene, cell cycle, cancer

## Abstract

γ-terpinene, α-terpinene, p-cymene, and myrcene are monoterpenes found in many essential oils extracted from a variety of plants and spices. Myrcene also occurs naturally in plants such as hops, cannabis, lemongrass, and verbena and is used as a flavoring agent in food and beverage manufacturing. In this research, the biological efficacy of γ-terpinene, α-terpinene, p-cymene, and myrcene was studied in human cell lines (HeLa, SH-SY5Y, and HDFa). Cytotoxicity, cell proliferation, cell migration, and morphology assays were performed to obtain detailed information on the anticancer properties. Our results show that myrcene has potential biological activity, especially in HeLa cells. In this cell line, it leads to an arrest of proliferation, a decrease in motility and morphological changes with loss of sphericity and thickness, and DNA damage. In addition, the interaction of γ-terpinene, α-terpinene, p-terpinene, and myrcene with calf thymus DNA (ct-DNA) was studied by UV-visible spectrophotometry. DNA binding experiments show that only myrcene can interact with DNA with an apparent dissociation constant (*K*_d_) of 29 × 10^−6^ M.

## 1. Introduction

In recent years, people have become increasingly sensitive to their quality of life, which is why the demand for renewable, natural and biodegradable resources has increased. This attention ranges from nutrition to medicine, as people’s awareness of the connection between food, medicinal agents, and healthy living has increased.

Plants play an important role in maintaining human health and improving quality of life, as they contain many phytochemicals that can be used in new biological medicines for pharmaceutical applications or are responsible for classifying foods as “functional”. Phytochemicals such as alkaloids, phenolic compounds, flavonoids, tannins, flavones, and terpenes are present in aromatic plants and spices, which is why these plants have long been used as anti-inflammatory, carminative, antioxidant, anticancer, and antiseptic agents [1].

In this context, terpenes are one promising source of natural products. One of the most known and studied class of terpenes are the monoterpenes, formed by two isoprene skeletons with 10 carbons derived from the mevalonate pathway. Monoterpenes are known for their enormous diversity, abundance, and remarkable biological properties, and the most studied monoterpenes are limonene, carvone, carveol, and perillyl alcohol, especially with regard to their chemotherapeutic activities [2].

Interestingly, a variety of dietary monoterpenes possess antitumor activity in animal models or various cell lines by preventing the development and progression of malignant tumors, also causing regression of pre-existing tumors [3,4]; human clinical trials are currently being conducted on these aspects. Inhibition of tumor cell proliferation, acceleration of tumor cell death, and induction of tumor cell differentiation are the main pathways of the cancer-suppressing activity of monoterpenes [2,5].

Monoterpenes also exhibit antioxidant properties, especially in relation to the oxidation of low-density lipo-proteins [6,7] and the inhibition of lipid peroxidation [8]. In addition, as antioxidants, monoterpenes can support the activity of other lipid-soluble or water-soluble antioxidants to provide superadditive/synergistic protection [9] or to enhance the efficacy of antioxidants such as phenols or aromatic amines [10].

Many studies aiming to investigate the antitumor activity of monoterpenes have been performed on essential oils extracted from a variety of plants, rather than on their individual components. The specific advantage of essential oils resides in the synergistic effects exerted by their components, compared with the sum effects of the individual substances [11].

Nonetheless, it is also important to clarify the biological activity of the individual monoterpenes, as carried out in a study that compared the cytotoxic effect of six monoterpenes (carvacrol, thymol, carveol, carvone, eugenol, and isopulegol) as well as their molecular mechanisms [12]. In this regard, Amorati et al. [13] recently investigated the unusual antioxidant activity of γ-terpinene that, after H-atom abstraction, forms an unstable peroxyl radical that breaks down, yielding para-cymene and a hydroperoxyl (HOO•) radical. This radical can donate an H-atom with a very high rate constant to alkylperoxyl radicals (ROO•), to the radicals of an antioxidant, or to ortho- and para-quinones.

**Figure 1:**
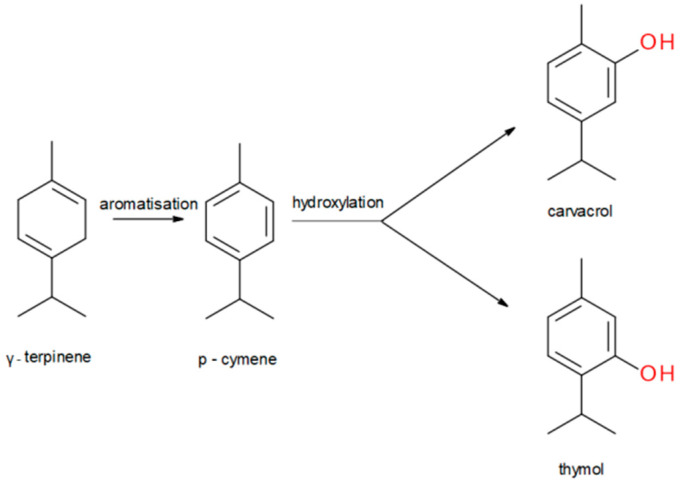
Scheme of the key steps of thymol and carvacrol biosynthesis [15].

γ-terpinene (γ-T), α-terpinene (α-T), p-cymene (p-cym), and myrcene (myr) are monoterpenes present in many essential oils extracted from a variety of plants and spices. γ-T and p-cym are the precursor molecules for both thymol and carvacrol (Figure 1), the two main components of thyme oil [14].

**Figure 2:**
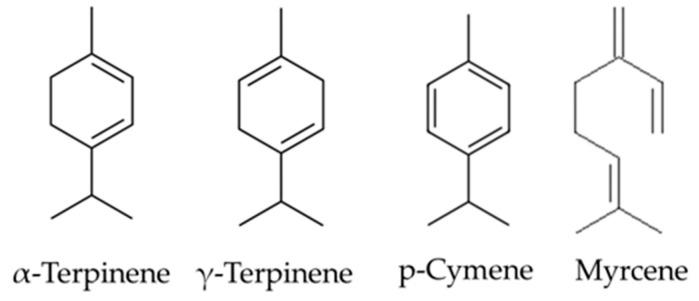
Structures of α-terpinene, γ-terpinene, p-cymene, and myrcene [16].

There is much less research on γ-terpinene, α-terpinene, p-cymene, and myrcene as individual molecules compared to thymol and carvacrol, and few studies linking these compounds to cytotoxicity activity; therefore, to broaden the previous chemical investigation to the biological one, our study focuses on the biological activity and the potential anticancer properties of these monoterpenes (Figure 2).

γ-T (1-isopropyl-4-methyl-1,4-cyclo-hexadine) has been shown to present some cytotoxic properties in the Jurkat cell line [17], human lung carcinoma A-549, and colon adenocarcinoma DLD-1 cells [18].

α-T (1-isopropyl-4-methyl-1,3-cyclo-hexadine) is a monoterpene found in the essential oils of a large variety of useful and aromatic plants such as tea tree, chenopodium, and murraya. Tea tree oil, containing α-terpinene and γ-terpinene among its main constituents, showed a remarkable antitumor activity, decreasing cell viability and cell proliferation of MCF-7 and 4T1 cells [19].

p-cym (1-methyl-4-(propan-2-yl)benzene) antitumoral effects were reported in the colorectal cancer rat model, and this property was linked to its antioxidant and anti-inflammatory effects [20]. p-cym is used also as a ligand for ruthenium, which gives rise to a number of organometallic compounds with promising anticancer properties [21].

Myr (7-Methyl-3-methylideneocta-1,6-diene) also occurs naturally in plants such as hops, cannabis, lemongrass, and verbena, and it is used as a flavoring agent in food and beverage manufacturing, while in brewing is one of the most potent aromatic flavor components. Several studies have shown that myr has anticarcinogenic potential in in vitro models. The cytotoxic effect of myr was found against a wide range of cancer cells, such as MCF-7 breast cancer [22], HT-29 colon adenocarcinoma and P388 leukemia cells [23], lung cancer cells [24], and other tumor cell lines [16,25]. Interestingly, myr ameliorated skin aging by decreasing the production of ROS, IL-6, and MMP-1 and MMP-3 (metalloproteinases) in UV-irradiated human skin fibroblasts [26].

In the present study, the cytotoxic activity of γ-T, α-T, p-cym, and myr and their potential influence on cell proliferation, random migration, and morphology were investigated in two cancer cell lines, namely, SH-SY5Y (human neuroblastoma) and HeLa (female cervical carcinoma), using HDFa (human primary dermal fibroblasts) as non-transformed control cells. Our results show that myr has potential anticancer biological activity, particularly in HeLa cells.

## 2. Results and Discussion

### 2.1. Cell Viability

**Figure 3:**
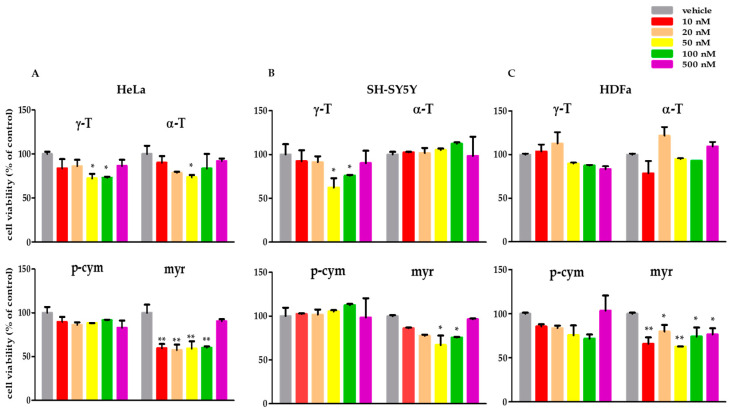
Cell viability of human malignant and non-malignant cells analyzed with MTT viability assay. The cells were treated for 24 h with increasing concentrations (10–500 nM) of γ-T, α-T, p-cym, and myr dissolved in DMSO (vehicle control). (**A**) HeLa, (**B**) SH-SY5Y, and (**C**) HDFa. Error bars are standard deviations. Significant differences are indicated as * *p* < 0.05, ** *p* < 0.01, significantly different from control (vehicle).

Two cancer cell lines, HeLa and SH-SY5Y, and one non-transformed line, HDFa, were exposed to each compound for 24 h, then potential cytotoxic activity was assessed using MTT assay. The results summarized in Figure 3 show that the compounds, when active, significantly decrease cell viability at very low concentrations between 10 and 50 nM. In HeLa cells, treatment with 50 and 100 nM γ-T decreases cell viability by about 25%; the same reduction was obtained with 50 nM α-T, whereas p-cym produces no significant effects. In these cells, myr proved to be the most potent compound, decreasing the viability of HeLa cells by about 40% when added at concentrations ranging between 10 and 100 nM (Figure 3A). Similar results were obtained in SH-SY5Y treated with 50–100 nM γ-T or myr, whereas the presence of α-T or p-cym was ineffective (Figure 3B). HDFa treatment with γ-T, α-T, and p-cym does not induce significant effects on cell viability, whereas the presence of myr at concentrations ranging from 10 to 500 nM causes a decrease in cell viability of about 20–30% (Figure 3C). Cell viability was also tested in the range 1–100 µM, but no effect was observed at these concentrations, probably owing to the high hydrophobicity of these molecules that tend to aggregate, limiting their capacity to enter the cells. Since the results obtained from the cell viability analysis show that the most active concentration for all the compounds tested is 50 nM, this concentration was chosen for all subsequent experiments.

### 2.2. Effects on Cell Proliferation, Motility, and Morphology

The biological effects of γ-T, α-T, p-cym, and myr were studied in HeLa, SH-SY5Y, and HDFa cell lines using Livecyte’s ptychography technology, which allows the simultaneous correlation of treatment-dependent changes in proliferation, motility, and cell morphology.

The effects on cell proliferation were analyzed using duplication time (DT) and the measurement of total dry mass (TDM) as parameters. In order to maintain a proper molecular composition, cells harmonize the activity of biosynthetic and catabolic pathways as well as the uptake and secretion of components. In proliferating cells, these processes are coordinated, and consequently all cellular components are doubled during each cell cycle, whereas differentiating cells may change their composition to match their own functions. Changes in molecular composition are frequently studied with approaches such as mass spectrometry, which can quantify the molecular details of a cell population after lysis. However, non-invasive methods, which can monitor the cellular composition of every single living cell with high resolution, are better suited for the study of dynamic and temporary events such as mitosis [27,28]. Cellular dry mass can be monitored using quantitative phase imaging (QPI), and this measurement is very important for understanding cell size and growth regulation [29,30,31].

**Figure 4:**
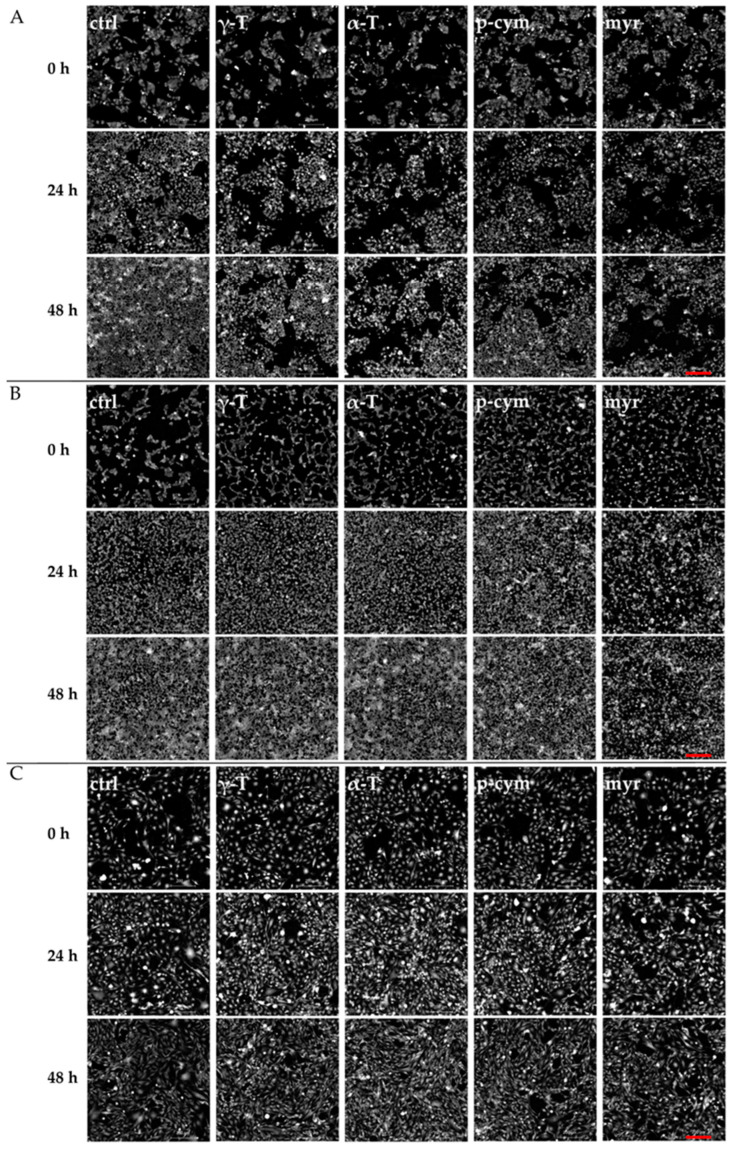
Quantitative phase imaging (QPI). Representative images of HeLa (**A**), SH-SY5Y (**B**), and HDFa (**C**) cells acquired at 0 h, 24 h, and 48 h of incubation with 50 nM of γ-T, α-T, p-cym, and myr. Scale bars: 200 µm.

The images of the cells reported in Figure 4 were obtained after 24 and 48 h of treatment with γ-T, α-T, p-cym, and myr. They confirm the MTT results, indicating that the most evident cytotoxic effects were obtained with myr in HeLa cells.

**Figure 5:**
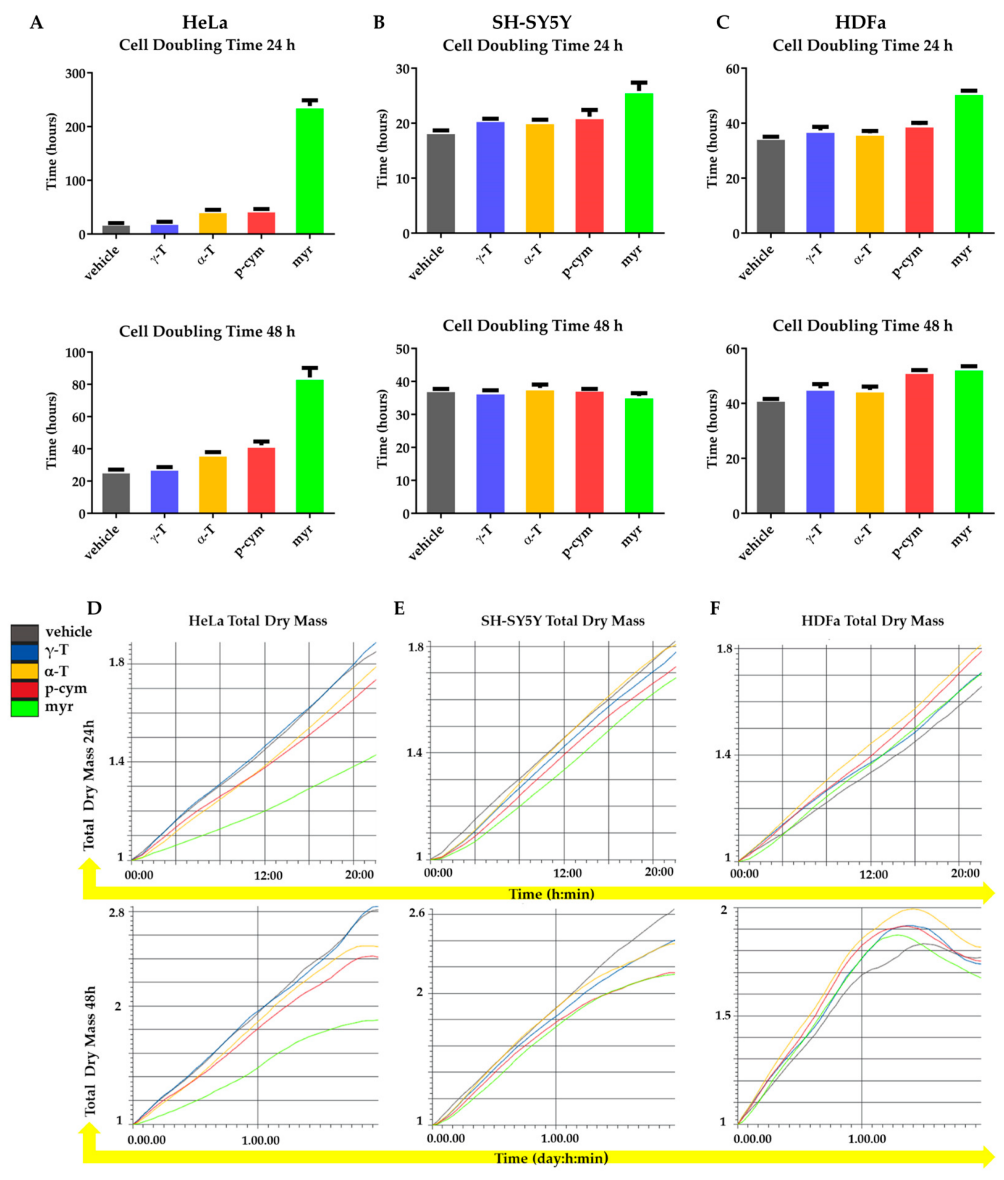
Effect of γ-T, α-T, p-cym, and myr treatment on cell proliferation. Cells have been treated (or not) with 50 nM compounds for 24 or 48 h. Above: histogram plot illustrates median cell doubling time for HeLa (**A**), SH-SY5Y (**B**), and HDFa (**C**) cells. Below: panels illustrate total dry mass for HeLa (**D**), SH-SY5Y (**E**), and HDFa (**F**). The different line colors correspond to the legend.

As shown in Figure 5B,C,E,F, treatment of SH-SY5Y or HDFa cells with γ-T, α-T, and p-cym for 24 or 48 h does not induce changes in DT or TDM. In contrast, 24 h treatment with myr in both SH-SY5Y and HDFa-cells results in an increase in DT (from 19 to 26 h and from 36 to 56 h, respectively), although these differences were attenuated after 48 h of treatment. The same condition does not cause any change in TDM of the two cell lines.

In HeLa cells, treatment with 50 nM γ-T for 24 or 48 h does not result in changes in DT or TDM. In contrast, treatment with 50 nM α-T or p-cym for 24 h induces an increase in DT from 20 to 42 h and from 20 to 46 h, respectively. After 48 h of treatment, p-cym still induces an increase in DT from 26 to 44 h. No changes in TDM are observed under these conditions at both 24 and 48 h (Figure 5A,D).

Myr is still the most active molecule. Indeed, an increase in DT from 20 to 269 h and from 26 to 87 h, respectively, is observed for both 24 and 48 h of treatment with 50 nM myr (Figure 5A). At the same time, a significant decrease in TDM is observed, as shown in Figure 5D.

The random cell migration assay requires analysis of a single cell and provides more in-depth information about cell speed and displacement. Cell migration is a highly coordinated event that plays a central role in a variety of physiological and pathological processes, including embryonic development [32,33], wound healing [34,35,36], and cancer metastasis [37,38,39].

In this study, we sought to directly quantify single-cell motility over 24 and 48 h of treatment. Automated measurement of single-cell migration was performed using Livecyte’s label-free QPI.

**Figure 6:**
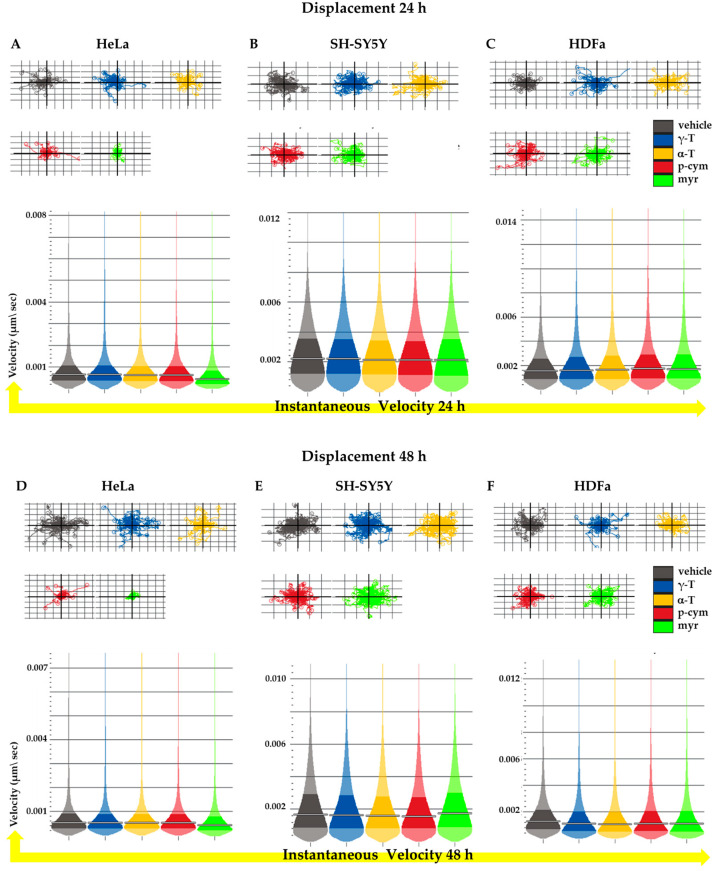
Analysis of cellular motility. (**Above**) Average confinement ratio of HeLa (**A**,**D**), SH-SY5Y (**B**,**F**), and HDFa (**C**,**F**) cells upon 24 or 48 h of treatment with different compounds at the concentration of 50 nM. Average velocity of HeLa (**A**,**D**), SH-SY5Y (**B**,**E**), and HDFa (**C**,**F**) cells upon 24 or 48 h treatment. Error bars represent the inter-quartile range.

For the SH-SY5Y and HDFa cell lines, treatment with the different compounds for 24 and 48 h does not result in significant changes in the displacement and velocity parameters, as shown in Figure 6B,C,E,F. In contrast, in HeLa cells, treatment with 50 nM p-cym or myr for 24 h decreases both migration and cell velocity, but only myr alters both parameters even after 48 h, whereas p-cym affects displacement but not velocity (Figure 6A,D).

**Figure 7:**
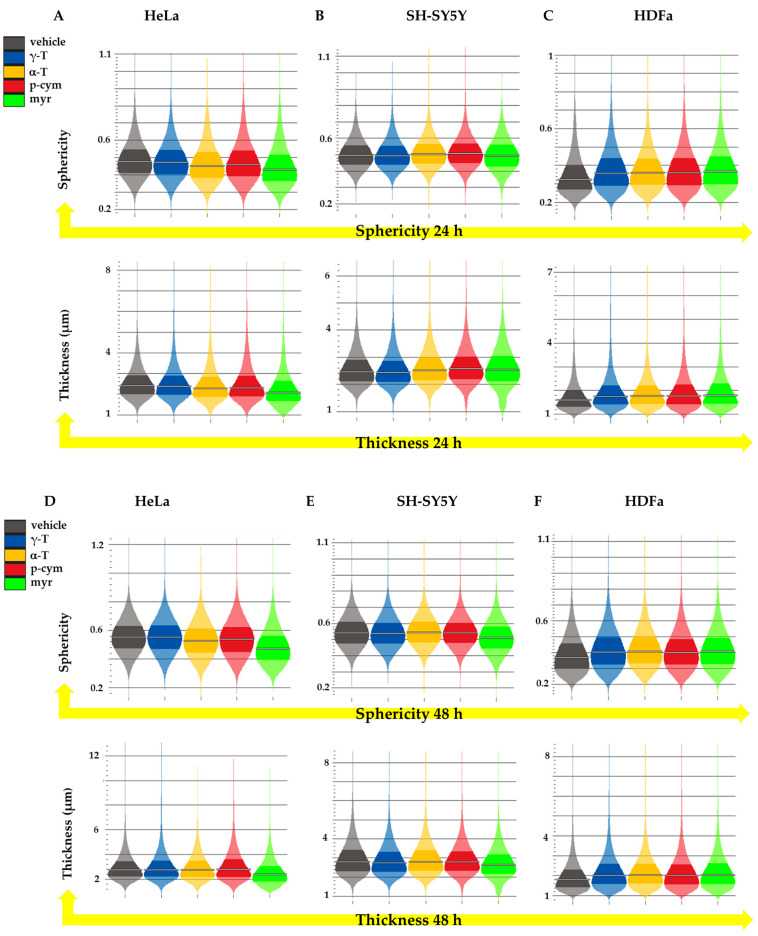
Analysis of cellular morphology. (**Above**) Average sphericity of HeLa (**A**,**D**), SH-SY5Y (**B**,**E**), and HDFa (**C**,**F**) cells upon 24 or 48 h of treatment with different compounds at the concentration of 50 nM. (**Below**) Average thickness of HeLa (**A**,**D**), SH-SY5Y (**B**,**E**), and HDFa (**C**,**F**) cells upon 24 or 48 h treatment. Error bars represent the inter-quartile range.

Cell morphology is assessed by analyzing the parameters of sphericity and thickness. The most significant changes were observed in HeLa cells treated with myr. Figure 7A,D shows that the addition of 50 nM myr reduced both sphericity and thickness of these cells after 24 and 48 h of treatment.

These data indicate that the prolonged doubling times observed in SH-SY5Y and HDFa cells after 24 h of treatment with myr could be due to a decreased proliferation rate or increased cell death without any temporal consequences. On the contrary, in HeLa cells, myr treatment induces more impressive biological effects by altering important cellular parameters such as DT, TDM, migration, and cell morphology in a sustained manner.

Our data confirm the observation reported in the literature, according to which the cytotoxic effects induced by these monoterpenes against the target cell lines reveal a different activity from one cell line to another, and a different sensitivity of each cell line to these compounds [12]. However, among the tested compounds, myr is the most active, and HeLa cells are the most sensitive target.

Very few data are reported in the literature concerning the cytotoxic effect or anticancer activity of myr. Silva and colleagues [25] investigated the cytotoxicity of myr against Vero (monkey kidney), A-549 (human lung carcinoma), HT-29 (human colon adenocarcinoma), and HeLa cell lines, as well as mouse macrophages. They found myr completely inactive against the tested cells, but the concentrations used were in the range of 1.6 µM to 1.6 mM, much higher than those used in the present study. Ferraz et al. [16] reported a cytotoxic activity for myr against HepG2 (human hepatocellular carcinoma) and B16-F10 (mouse melanoma) cell lines, with an IC_50_ value ranging between 74 and 98 µM. Bai and Tang [24] observed a cytotoxic effect against A549 human lung carcinoma cells upon the addition of 2–8 µM myr. In our hands, myr concentrations higher than 500 nM failed to exert a cytotoxic effect in any type on tested cells, probably due to the tendency of this molecule to aggregate.

Subsequent studies were designed to clarify whether the myr-induced antiproliferative activity could be due to a delay in a specific phase of the cell cycle.

To this purpose, HeLa cells were treated with 50 nM myr for 24 h, then stained with PI and subjected to flow cytometric analysis.

**Figure 8:**
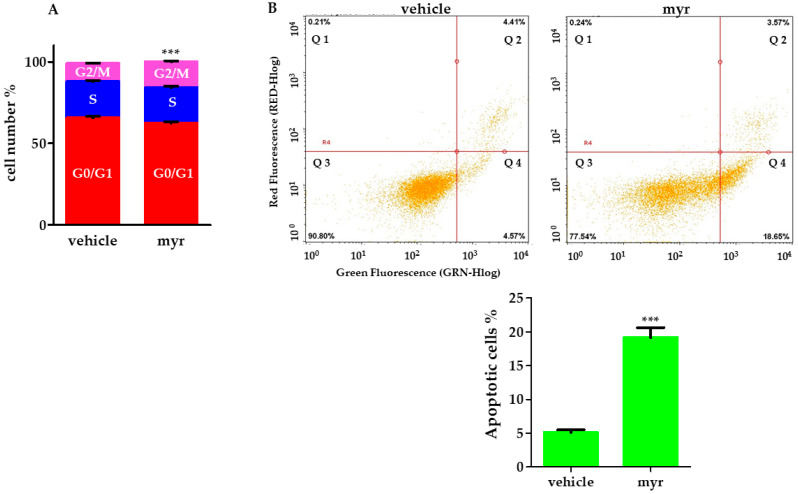
Flow cytometric analysis of cell cycle and apoptosis. (**A**) HeLa cells were treated with 50 nM myr for 24 h and then analyzed using flow cytometry. Error bars are standard deviations. Significant differences are indicated by *** *p* < 0.001 significantly different from control (vehicle). (**B**) Top. Representative images of dot plots of annexin V-FITC/PI. Cytofluorimetric analysis of annexin V-FITC- and PI-stained cells in vehicle (control) and after 24-h treatment with 50 nM myr. Four quadrants represent Q1 necrotic cells (annexin V/negative; PI/positive), Q2 late apoptotic cells (annexin V/positive; PI/positive), Q3 viable cells (annexin V/negative; PI/negative), and Q4 early apoptotic cells (annexin V/positive; PI/negative). Numbers indicate the percentage of cells in each quadrant, and at least 5000 events were read (*n* = 3). Bottom. Quantitative analysis of cell apoptosis with annexin V/PI double staining in flow cytometry. Data were presented as mean ± SD of three independent experiments performed in triplicate. *** *p* < 0.001, compared with vehicle.

Figure 8A reports that myr-treated HeLa cells show a significant decrease in G0/G1 followed by an accumulation in G2/M phase, approximately by 30.4% ± 4.5.

To further investigate the effects of myr on cell death and to clarify whether it can be considered an apoptosis inducer, HeLa cells were stained with annexin V and propidium iodide (PI) after 24 h of treatment. Externalized phosphatidylserine (PS) not only contributes to the recognition and subsequent removal of apoptotic bodies by phagocytes [40,41], but it also provides a binding site for the anionic lipid-binding protein annexin V [42], which is commonly used to detect apoptotic cells [43]. PI staining indicates a change in membrane integrity that occurs upon necrosis or subsequent apoptosis.

Figure 8B shows the histogram with dual parameters, including annexin V-FITC and PI. HeLa cells stained positive for annexin V and negative for propidium iodide showed a significant increase from 4.75% to 18.65% after 24-h exposure to myr (Figure 8B).

These data are in agreement with those obtained by Bai and Tang [24], who reported that the ability of myrcene to block the cell cycle or induce apoptosis is one of the mechanisms through which this compound exerts its anticancer activity.

### 2.3. Myrcene Induced DNA Damage in HeLa Cells

The H2A histone family comprises three subfamilies whose members contain characteristic sequence elements that have been independently conserved during eukaryotic evolution. The three H2A subfamilies are H2A1-H2A2, H2AZ, and H2AX; in mammals, H2AZ accounts for approximately 10% of the H2A complement, while H2AX accounts for 2–25%, and H2A1-H2A2 represents the remainder [44].

When DNA is damaged, indifferently by endogenous or exogenous agents, double-strand breaks (DSBs) are formed, always followed by phosphorylation of histone H2AX at serine 139, identified as γH2AX. This histone is phosphorylated by kinases such as ataxia telangiectasia mutated (ATM) and ATM-Rad3-related (ATR) following the PI3K pathway [45].

Cell-cycle checkpoints help to ensure the accuracy of DNA replication and division. These checkpoints allow progression through the cell cycle or arrest in response to DNA damage in order to give time for DNA repairing. The cell-cycle DNA damage checkpoints occur late in G1, which prevents entry in S phase, and late in G2, which prevents entry in mitosis [46,47]

**Figure 9:**
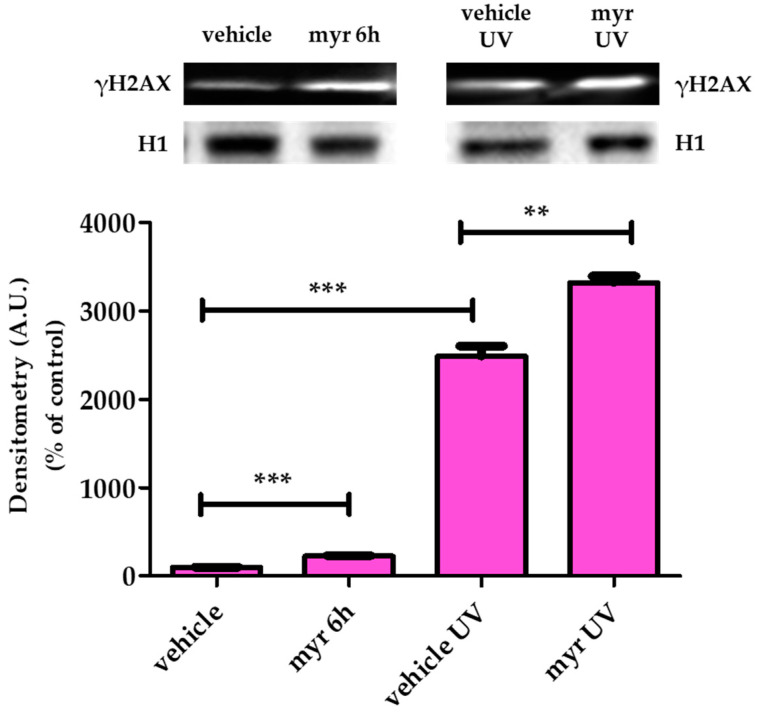
Myr induces DNA damage in HeLa cells. (Above) Representative Western blot image of γH2AX in HeLa cells treated with myr (50 nM), exposed to UV, or treated with myr and UV for 6 h. (Below) Relative quantification. Arbitrary densitometry units (A.U.) were normalized by H1 histone. All data represent mean ± SD (*n* = 6). ** *p* < 0.01, *** *p* < 0.001, significantly different from control (vehicle).

Therefore, we studied the effect of myr on DNA damage by evaluating the phosphorylation status of H2AX in Western blot. UV-irradiated HeLa cells were used as a positive control. Cells were treated with vehicle or 50 nM myr for 6 h, then histones were extracted and analyzed for the presence of γH2AX. As shown in Figure 9, myr and UV treatment significantly increase γH2AX levels compared with the untreated control.

HeLa cells treated with myr were exposed to UV light. Interestingly, γH2AX significantly increases compared to the UV-treated control, indicating that myr increases UV cytotoxicity (Figure 9). Similar results were obtained by Bai and Tang [24], who demonstrated that A549 lung adenocarcinoma cells exposed to myrcene exhibited a significant increase in γH2AX phosphorylation, indicating that DNA damage is activated by this monoterpene.

DNA-damaging agents are widely used in oncology to treat both hematological and solid cancers. Some commonly used modalities include ionizing radiation, platinum drugs (cisplatin, oxaliplatin, and carboplatin), cyclophosphamide, chlorambucil, and temozolomide [48,49]. By modifying the chemical structure of nucleic acids, these agents induce cytotoxicity and subsequently elimination of cancer cells from the body.

In order to compare the binding properties of the compounds with DNA, dissociation constants (*K*_d_) were determined through inverse titration experiments.

The increase in the differential absorption of DNA in the presence of γ-T, α-T, or p-cym can be ascribed to a lower base stacking, while the decreased differential absorbance observed for myr suggests a higher compactness of DNA.

**Figure 10:**
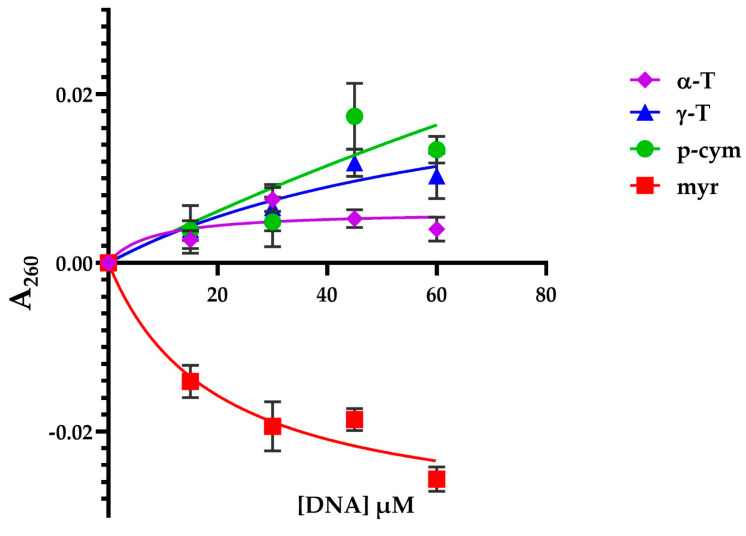
Differential absorbance at 260 nm of DNA-molecule complexes at increasing DNA concentration. Molecule concentration is 50 nM.

The differential absorbance at 260 nm for each molecule versus DNA concentration was plotted, as reported in Figure 10. The estimation of the dissociation constant (*K*_d_) for the complex formation was obtained by fitting the data using a one-site saturation equation (Table 1).

*K*_d_ analysis confirms that myr interacts with DNA (*K*_d_ = 29 ± 18 μM), suggesting a partial explanation for its cellular toxicity. Since the SE and r^2^ values for the other terpenes are very high, we considered the corresponding *K*_d_ values as non reliable. The decrease in absorbance at 260 nm observed in the presence of myr could be indicative of DNA supercoiling.

## 3. Materials and Methods

### 3.1. Cell Culture and Treatments

HeLa human cervix adenocarcinoma cells, SH-SY5Y human neuroblastoma, and HDFa human normal fibroblast as control cell lines were purchased from American Type Culture Collection (ATCC, Manassas, VA, USA). Cells were cultured in RPMI 1640 medium (Labtek Eurobio, Milan, Italy), supplemented with 10% FCS (Euroclone, Milan, Italy) and 2 mM L-glutamine (Sigma-Aldrich, Milan, Italy), at 37 °C, and a 5% CO_2_ atmosphere. γ-T, α-T, p-cym, and myr were purchased from Sigma-Aldrich (Sigma-Aldrich, Milan, Italy). γ-T, α-T, p-cym and myr were percolated twice through activated basic alumina and once through silica to remove impurities and traces of hydroperoxides. The compounds were dissolved in DMSO in a 30 mM stock solution. In cell treatments, the final DMSO concentration never exceeded 0.1%.

#### 3.1.1. MTT Assay

Cells were seeded at 1.5 × 10^4^ cells/well in a 96-well culture plastic plate (Sarsted, Milan, Italy), and after 24h growth exposed to increasing concentrations of γ-T, α-T, p-cym, and myr (from 0.010 μM to 100 μM) solubilized in RPMI 1640 medium. After 24 h of treatment, the culture medium was replaced with 0.1 mL of 3-(4,5-dimethylthiazolyl-2)-2,5-diphenyltetrazolium bromide (MTT), (Sigma-Aldrich, Milan, Italy) dissolved in PBS at the concentration of 0.2 mg/mL, and samples were incubated for 2 h at 37 °C. The absorbance at 570 nm was measured using a multi-well plate reader (Tecan, Männedorf, CH), and data were analyzed using Prism GraphPad software (GraphPad Software, version 6.0, San Diego, CA, USA) and expressed as IC_50_ μM.

#### 3.1.2. Quantitative Phase Image Microscopy

Quantitative phase image (QPI) microscopy assay was performed using a Livecyte microscope (Phase Focus Limited, Sheffield, UK) according to the manufacturer’s indications. In brief, HeLa, SH-SY5Y, and HDFa cells were seeded in a 96-well plate (Sarsted, Milan, Italy) at 4 × 10^3^ per well. After 24 h, cells were treated with 50 nM γ-T, α-T, p-cym and myr in six replicates and the images acquired every 60 min for 2 days using a 10× objective (0.25 NA), at 37 °C and 5% CO_2_. Data were analyzed using the Cell Analysis Toolbox software, (version 3.10.0, Phase Focus Limited, Sheffield, UK) to evaluate cell growth, doubling times, and motility.

#### 3.1.3. Cell-Cycle and Apoptosis Analysis Using Flow Cytometry

HeLa cells were plated at a density of 2 × 10^3^ cell/cm^2^ in a dish and after 24 h treated with 50 nM myr for 24 h. Untreated and treated cells were detached, washed in PBS, and the pellet suspended in 0.01% NP-40 (Sigma-Aldrich, Milan, Italy), 10 μg/mL RNase (Sigma-Aldrich, Milan, Italy), 0.1% sodium citrate (Sigma-Aldrich, Milan, Italy); then, 50 μg/mL propidium iodide (PI) (Sigma-Aldrich, Milan, Italy) was added and the suspension maintained for 30 min at room temperature in the dark. Flow cytometric assays were performed on Bio-Rad S3 flow cytometer (Bio-Rad, Watford, UK) equipped with an argon ion laser; PI fluorescence was collected at 600 nm on a linear scale.

For apoptosis analysis, HeLa cells were plated out at a density of 2 × 10^3^ cells/cm^2^ in a dish and treated with 50 nM myr for 24 h. Then, cells were analyzed to determine cell apoptosis using an annexin V-FITC/PI Apoptosis Detection Kit (Elabscience, Houston, TX, USA). The population of apoptotic cells was determined and analyzed using the Guava easyCyte 5HT flow cytometer (Luminex Corporation, Austin, TX, USA) with an excitation wavelength of 488 nm and emission at 525 nm (FITC fluorescence) and 600 nm (PI fluorescence) on a logarithmic scale.

#### 3.1.4. Histone Post-Translational Modification

HeLa cells were seeded in a dish and after 72 h treated for 6 h with myr at a final concentration of 50 nM. As a positive control, cells were treated with UV radiation that induces global DNA damage. Cells were cultured as above and after 48 h irradiated with a 7.5-Watt UV lamp for 3 min. Cells were harvested, washed with 10 mM sodium butyrate in PBS, and nuclei isolated according to Micheletti [50]. In brief, the nuclear pellet was suspended in 0.1 mL ice-cold H_2_O, and concentrated H_2_SO_4_ was added to the suspension to give a final concentration of 0.4 N. After incubation at 4 °C for 1 h, the suspension was centrifuged for 5 min at 14,000× *g*, and the supernatant taken and mixed with 1 mL of acetone. After overnight incubation, the coagulate material was collected via microcentrifugation and air-dried. This histone fraction was dissolved in H_2_O. Proteins were quantified using a protein assay kit (Bio-Rad, Hercules, CA, USA). Histones were separated on a 15% gel in Tris–glycine buffer using electrophoresis at 150 V for 90 min, then transferred to a nitrocellulose membrane. The nitrocellulose membrane was incubated with rabbit anti γH2AX (Santa Cruz, CA, USA) primary antibody for 1 h. After washing with PBS-TWEEN 20 0.1%, the membrane was incubated with rabbit secondary HRP-conjugated antibodies (GE Healthcare, Milan, Italy). After washing with PBS-TWEEN 20 0.1%, the antibody binding was detected using Amersham ECL Plus Western Blotting Detection System (GE Healthcare, Milan, Italy). Densitometric analysis was performed using a Fluor-S Max MultiImager (Bio-Rad, Hercules, CA, USA), and relative quantification of histone acetylation signals was performed using densitometry and normalized on an H1 signal as a control.

#### 3.1.5. DNA–Compound Interaction Assay

Absorption titration experiments were carried out by keeping constant the concentration of γ-T, α-T, p-cym, and myr (50 nM) while raising the DNA concentration from 15 μM to 60 μM in a buffer solution (BS; 50 mM NaCl, 5 mM TRIS buffer, pH 7.2) and 0.8% of DMSO. The UV-Vis spectra were obtained using a Jasco V-550 spectrophotometer (Jasco, Hachioji-shi, Japan). The absorbance spectra were obtained scanning the solution in 1 cm quartz cuvettes from 235 nm to 310 nm using a 2 nm bandwidth. Then, additions of standard DNA were performed, and after each addition of DNA, the absorbance spectrum was recorded. The DNA stock solution was prepared at low molecular weight from salmon sperm (Sigma-Aldrich, Milan, Italy) in BS. The DNA stock solution concentration was determined spectrophotometrically (*λ*: 260 nm) using an extinction coefficient of 6600 M^−1^ cm^−1^.

The blanks were prepared with standard DNA titration from 15 μM to 60 μM in BS and 0.8% of DMSO.

The UV-Vis spectra of DNA in the presence of the different compounds were obtained using a Jasco V-550 spectrophotometer. The data analysis was carried out on the subtracted spectrum (ΔA_λ_): the spectra of titration experiments were subtracted using standard DNA titration and compounds lacking DNA:

ΔA_λ_ = (ΔA_DNA − compound_)_λ_ − (ΔA_DNA_)_λ_ − (ΔA_compound_)_λ_

Compound absorbance variations at 260 nm (ΔA_260_) were plotted versus DNA concentration. The calculation of binding parameters was carried out fitting ΔA_260_ using the following equation:

$$\Delta A_{260}=\frac{B_{max}[DNA]}{K_{d}+[DNA]}$$

where *K*_d_ represents the dissociated constant of DNA complex for compounds, and *B*_max_ represents the limiting value of ΔA_260_ [51].

### 3.2. Statistical Analysis

A two-way ANOVA corrected for multiple comparisons (Dunnett test) was performed with GraphPad Prism software (GraphPad Software, version 6.0, San Diego, CA, USA). All treatments were analyzed against control (DMSO). Significance was graphically reported as follows: * *p* < 0.05, ** *p* < 0.01, and *** *p* < 0.001.

## 4. Conclusions

In this paper we have evaluated the biological activity of four monoterpenes, γ-T, α-T, p-cym, and myr, on two different cancer cell lines (SH-SY5Y and HeLa) and a normal fibroblast (HDFA).

Among them, only myr possesses a potent biological activity in HeLa cells. In fact, this molecule, at the concentration 50 nM, inhibits proliferation by increasing DT and significantly modifies cell motility, indicating that cells are arrested in growth and that the capacity for invasion is strongly inhibited. In HeLa cells, growth arrest in the G2/M phase of the cell cycle was associated with increased apoptotic death and phosphorylation of histone H2AX (γ-H2AX), indicating DNA damage.

These effects could be related to an interaction of myr with DNA, and this hypothesis is supported by the results of DNA titration, in which the dissociation constant of myr with salmon sperm DNA is 29.03 µM. The exact mechanism of myrcene-DNA interaction has not been fully elucidated, but studies are underway in our laboratory to elucidate the structure–activity relationship. Finally, UV irradiation of myr-treated cells enhances DNA damage, suggesting that this combined treatment could be a powerful therapy to target chemotherapy- and radiotherapy-resistant tumors.

## Figures and Tables

**Table 1:** Dissociation constant for compounds formation for the ΔA_260_ obtained by fitting data in Figure 10.

Compound	*K*_d_ ± SE (μM)	r^2^
γ-T	74 ± 83	0.8316
α-T	17 ± 22	0.7311
p-cym	493 ± 4425	0.6339
myr	29 ± 18	0.9360

## Data Availability

Not applicable.

## Sample Availability

Samples of some compounds are available from the authors.
